# Supplementary material for: The Occurrence of Five Unregulated Mycotoxins Most Important for Traditional Dry-Cured Meat Products
Source: Toxins (Basel). 2022 Jul 12;14(7):476. doi: 10.3390/toxins14070476 (PMC9315684; doi:10.3390/toxins14070476)
Supplement: Supplementary file 1 [file toxins-14-00476-s001.zip › toxins-1778968-supplementary.pdf]

toxins

MDPI

Supplementary Materials: The Occurrence of Five Unregulated Mycotoxins Most Important for Traditional Dry-Cured Meat Products

Tina Lešić, Ana Vulić, Nada Vahčić, Bojan Šarkanj, Brigita Hengl, Ivica Kos, Tomaž Polak, Nina Kudumija and Jelka Pleadin

Table S1. The mycotoxins detected in traditional dry-cured meat products according to product type.

**Product group**		**Dry-fermented sausages**		**Dry-cured meats**			
**Product type**		**Domestic susages n=70**	***Kulen/Kulenova seka*** **n=38**	**Prosciutto/Dry ham n=41**	**Dry rack n=22**	*Pečenica/Ombolo* n=29	Bacon/pancetta n=50
STC	n% / n	7/5	5/2	< LOD	5/1	7/2	2/1
	Mean±SD (Min-max)	0.17±0.09 (0.10-0.29)	2.30±2.31 (0.66-3.93)	< LOD	0.12±na	0.15±0.02 (0.13-0.16)	0.49±na
	Median	0.12	2.30	< LOD	na	0.15	na
OTA	n% / n	19/13	16/6	2/1	5/1	3/1	2/1
	Mean±SD (Min-max)	0.82±0.78 (0.24-3.02)	0.81±0.50 (0.29-1.65)	4.81±na	2.66±na	0.49±0.22	0.68±na
	Median	0.51	0.78	na	na	0.49	na
CPA	n% / n	13/9	18/7	15/6	9/2	17/5	8/4
	Mean±SD (Min-max)	54.84±144.73 (3.10-335.50)	13.38±9.53 (5.80-27.25)	29.53±43.20 (4.20-108.50)	6.43±0.46 (6.10-6.75)	30.24±25.55 (6.25-66.35)	8.26±4.09 (3.20-12.80)
	Median	6.55	13.35	20.90	6.43	27.05	8.53

LOD—limit of detection; na—not applicable; n%/n—percentage/number of samples above LOD.
